# Supplementary material for: Antimicrobial Resistance in Rapidly Growing Nontuberculous Mycobacteria among Domestic and Wild Animals Emphasizing the Zoonotic Potential
Source: Microorganisms. 2023 Oct 9;11(10):2520. doi: 10.3390/microorganisms11102520 (PMC10609087; doi:10.3390/microorganisms11102520)
Supplement: Supplementary file 1 [file microorganisms-11-02520-s001.zip › microorganisms-2611464-supplementary-done.pdf]

microorganisms

MDPI

Article

Antimicrobial Resistance in Rapidly Growing Nontuberculous Mycobacteria among Domestic and Wild Animals Emphasizing the Zoonotic Potential

Irena Reil [1,†], Silvio Špičić [1,†], Ljubo Barbić [2], Sanja Duvnjak [1,*], Gordan Kompes [1,*], Miroslav Benić [1], Dora Stojević [1], Željko Cvetnić [1], Jurica Arapović [3,4] and Maja Zdelar-Tuk [1]

[1] Croatian Veterinary Institute, 10000 Zagreb, Croatia; irena.reil@gmail.com (I.R.); spicic@veinst.hr (S.Š.); benic@veinst.hr (M.B.); dstojevic@gmail.com (D.S.); cvetnic@veinst.hr (Ž.C.); zdelar-tuk@veinst.hr (M.Z.-T.)
[2] The Faculty of Veterinary Medicine, University of Zagreb, 10000 Zagreb, Croatia; ljbarbic@gmail.com
[3] Department of Infectious Diseases, University Clinical Hospital Mostar, 88000 Mostar, Bosnia and Herzegovina; jurica.arapovic@mef.sum.ba
[4] School of Medicine, University of Mostar, 88000 Mostar, Bosnia and Herzegovina
* Correspondence: marjanovic@veinst.hr (S.D.); kompes@veinst.hr (G.K.); Tel.: +385-16123635 (S.D.); +385-16123657 (G.K.)
† These authors contributed equally to this work.

Table S1. The results of determining the antimicrobial susceptibility of rapidly-growing NTM isolates.

No. in list	Animal species	NTM species	Interpretation of susceptibility test and MIC (µg/ml)														
			AMI	AUG2	FEP	FOX	AXO	CIP	CLA (5th day/ 14th day)	DOX	IMI	LZD	MIN	MXF	TGC	TOB	SXT
1	wild boar	*M. arupense*	≤ 1 (S)	16/8 (I)	16 (I)	≤ 4 (S)	32 (I)	≤ 0,12 (S)	0,12/ 0,12 (S/S)	≤ 0,12 (S)	8 (I)	≤ 1 (S)	≤ 1 (S)	≤ 0,25 (S)	0,3	≤ 1 (S)	≤ 0,25/ 4,75 (S)
2	wild boar	*M. arupense*	≤ 1 (S)	8/4 (S)	8 (S)	≤ 4 (S)	32 (I)	≤ 0,12 (S)	≤ 0,06/ ≤ 0,06 (S/S)	≤ 0,12 (S)	16 (I)	≤ 1 (S)	≤ 1 (S)	≤ 0,25 (S)	0,1	≤ 1 (S)	≤ 0,25/ 4,75 (S)
3	cattle	*M. chitae*	≤ 1 (S)	8/4 (S)	4 (S)	≤ 4 (S)	≤ 4 (S)	≤ 0,12 (S)	≤ 0,06/ 0,5 (S/S)	≤ 0,12 (S)	≤ 2 (S)	≤ 1 (S)	≤ 1 (S)	≤ 0,25 (S)	0,1	≤ 1 (S)	≤ 0,25/ 4,75 (S)
4	cattle	*M. chitae*	4 (S)	4/2 (S)	8 (S)	≤ 4 (S)	≤ 4 (S)	≤ 0,12 (S)	> 16 (5th day) (R)	≤ 0,12 (S)	≤ 2 (S)	2 (S)	≤ 1 (S)	≤ 0,25 (S)	0,5	≤ 1 (S)	≤ 0,25/ 4,75 (S)
5	cattle	*M. chitae*	≤ 1 (S)	32/16 (R)	4 (S)	8 (S)	≤ 4 (S)	≤ 0,12 (S)	4/4 (I/I)	≤ 0,12 (S)	16 (I)	≤ 1 (S)	≤ 1 (S)	≤ 0,25 (S)	0,5	≤ 1 (S)	≤ 0,25/ 4,75 (S)
6	cattle	*M. chitae*	≤ 1 (S)	64/32 (R)	4 (S)	≤ 4 (S)	≤ 4 (S)	≤ 0,12 (S)	4/16 (I/R)	≤ 0,12 (S)	4 (S)	≤ 1 (S)	≤ 1 (S)	≤ 0,25 (S)	0,3	≤ 1 (S)	≤ 0,25/ 4,75 (S)
No. in list	Animal species	NTM species	Interpretation of susceptibility test and MIC (µg/ml)														
			AMI	AUG2	FEP	FOX	AXO	CIP	CLA (5th day/ 14th day)	DOX	IMI	LZD	MIN	MXF	TGC	TOB	SXT
7	roe deer	*M. chitae*	≤ 1 (S)	≤ 2/1 (S)	4 (S)	8 (S)	≤ 4 (S)	≤ 0,12 (S)	0,25/ 0,25 (S/S)	≤ 0,12 (S)	≤ 2 (S)	≤ 1 (S)	≤ 1 (S)	≤ 0,25 (S)	0,5	≤ 1 (S)	≤ 0,25/ 4,75 (S)
8	roe deer	*M. chitae*	≤ 1 (S)	4/2 (S)	32 (R)	≤ 4 (S)	8 (S)	≤ 0,12 (S)	≤ 0,06/ ≤ 0,06 (S/S)	≤ 0,12 (S)	4 (S)	≤ 1 (S)	≤ 1 (S)	≤ 0,25 (S)	0,3	≤ 1 (S)	≤ 0,25/ 4,75 (S)
9	cattle	*M. elephantis*	≤ 1 (S)	16/8 (I)	8 (S)	≤ 4 (S)	≤ 4 (S)	0,25 (S)	1/1 (S/S)	0,25 (S)	> 64 (R)	≤ 1 (S)	≤ 1 (S)	≤ 0,25 (S)	0,3	4 (I)	≤ 0,25/ 4,75 (S)
10	cattle	*M. fortuitum*	≤ 1	16/8	> 32	32	> 64	2	0,25/1	4	NR	8	2	1	2	≤ 1	2/38

No. in list	Animal species	NTM species	Interpretation of susceptibility test and MIC (μg/ml)														
			AMI	AUG2	FEP	FOX	AXO	CIP	CLA (5th day/ 14th day)	DOX	IMI	LZD	MIN	MXF	TGC	TOB	SXT
			(S)	(I)	(R)	(I)	(R)	(I)	(S/S)	(I)		(S)	(I)	(S)		(S)	(S)
11	cattle	*M. fortuitum*	8<br>(S)	64/32<br>(R)	32<br>(R)	32<br>(I)	8<br>(S)	2<br>(I)	≤ 0,06/<br>0,25<br>(S/S)	4<br>(I)	NR	4<br>(S)	4<br>(I)	1<br>(S)	2	4<br>(I)	1/19<br>(S)
12	cattle	*M. fortuitum*	≤ 1<br>(S)	16/8<br>(I)	> 32<br>(R)	64<br>(I)	> 64<br>(R)	0,25<br>(S)	> 16<br>(5th day)<br>(R)	≤ 0,12<br>(S)	8<br>(I)	2<br>(S)	≤ 1<br>(S)	≤ 0,25<br>(S)	0,1	≤ 1<br>(S)	≤ 0,25/<br>4,75<br>(S)
13	cattle	*M. fortuitum*	≤ 1<br>(S)	32/16<br>(R)	> 32<br>(R)	128<br>(R)	> 64<br>(R)	0,25<br>(S)	> 16<br>(5th day)<br>(R)	≤ 0,12<br>(S)	≤ 2<br>(S)	2<br>(S)	≤ 1<br>(S)	≤ 0,25<br>(S)	0,3	≤ 1<br>(S)	≤ 0,25/<br>4,75<br>(S)
14	cattle	*M. fortuitum*	2<br>(S)	8/4<br>(S)	8<br>(S)	≤ 4<br>(S)	8<br>(S)	≤ 0,12<br>(S)	0,12/<br>0,12<br>(S/S)	0,5<br>(S)	≤ 2<br>(S)	4<br>(S)	≤ 1<br>(S)	≤ 0,25<br>(S)	0,5	≤ 1<br>(S)	≤ 0,25/<br>4,75<br>(S)
No. in list	Animal species	NTM species	Interpretation of susceptibility test and MIC (μg/ml)														
			AMI	AUG2	FEP	FOX	AXO	CIP	CLA (5th day/ 14th day)	DOX	IMI	LZD	MIN	MXF	TGC	TOB	SXT
15	domestic pig	*M. fortuitum*	2<br>(S)	64/32<br>(R)	> 32<br>(R)	64<br>(I)	> 64<br>(R)	2<br>(I)	4/16<br>(I/R)	> 16<br>(R)	NR	16<br>(I)	> 8<br>(R)	1<br>(S)	2	> 16<br>(R)	2/38<br>(S)
16	wild boar	*M. fortuitum*	2<br>(S)	32/16<br>(R)	> 32<br>(R)	≤ 4<br>(S)	64<br>(R)	≤ 0,12<br>(S)	4/8<br>(I/R)	2<br>(I)	8<br>(I)	2<br>(S)	≤ 1<br>(S)	≤ 0,25<br>(S)	0,3	2<br>(S)	≤ 0,25/<br>4,75<br>(S)
17	wild boar	*M. fortuitum*	≤ 1<br>(S)	32/16<br>(R)	16<br>(I)	8<br>(S)	32<br>(I)	≤ 0,12<br>(S)	4/4<br>(I/I)	0,5<br>(S)	8<br>(I)	≤ 1<br>(S)	≤ 1<br>(S)	≤ 0,25<br>(S)	0,1	≤ 1<br>(S)	≤ 0,25/<br>4,75<br>(S)
18	wild boar	*M. fortuitum*	≤ 1<br>(S)	8/4<br>(S)	16<br>(I)	16<br>(S)	64<br>(R)	≤ 0,12<br>(S)	4/>16<br>(I/R)	8<br>(R)	4<br>(S)	4<br>(S)	4<br>(I)	≤ 0,25<br>(S)	0,1	8<br>(R)	0,5/9,5<br>(S)
19	wild boar	*M. fortuitum*	2<br>(S)	32/16<br>(R)	> 32<br>(R)	16<br>(S)	> 64<br>(R)	≤ 0,12<br>(S)	8/8<br>(R/R)	4<br>(I)	8<br>(I)	2<br>(S)	2<br>(I)	≤ 0,25<br>(S)	0,3	8<br>(R)	0,5/9,5<br>(S)
20	wild boar	*M. fortuitum*	≤ 1<br>(S)	16/8<br>(I)	> 32<br>(R)	64<br>(I)	> 64<br>(R)	0,5<br>(S)	> 16<br>(5th day)<br>(R)	0,5<br>(S)	8<br>(I)	4<br>(S)	≤ 1<br>(S)	≤ 0,25<br>(S)	0,1	≤ 1<br>(S)	≤ 0,25/<br>4,75<br>(S)
21	wild boar	*M. fortuitum*	≤ 1<br>(S)	32/16<br>(R)	> 32<br>(R)	≤ 4<br>(S)	> 64<br>(R)	≤ 0,12<br>(S)	≤ 0,06/1<br>(S/S)	2<br>(I)	≤ 2<br>(S)	≤ 1<br>(S)	2<br>(I)	≤ 0,25<br>(S)	0,3	≤ 1<br>(S)	≤ 0,25/<br>4,75<br>(S)
22	roe deer	*M. fortuitum*	≤ 1<br>(S)	32/16<br>(R)	> 32<br>(R)	≤ 4<br>(S)	> 64<br>(R)	≤ 0,12<br>(S)	0,5/<br>0,5<br>(S/S)	≤ 0,12<br>(S)	≤ 2<br>(S)	≤ 1<br>(S)	≤ 1<br>(S)	≤ 0,25<br>(S)	0,3	2<br>(S)	≤ 0,25/<br>4,75<br>(S)
No. in list	Animal species	NTM species	Interpretation of susceptibility test and MIC (μg/ml)														
			AMI	AUG2	FEP	FOX	AXO	CIP	CLA (5th day/ 14th day)	DOX	IMI	LZD	MIN	MXF	TGC	TOB	SXT
23	roe deer	*M. fortuitum*	≤ 1<br>(S)	8/4<br>(S)	32<br>(R)	≤ 4<br>(S)	32<br>(I)	≤ 0,12<br>(S)	0,25/<br>0,5<br>(S/S)	≤ 0,12<br>(S)	8<br>(I)	≤ 1<br>(S)	≤ 1<br>(S)	≤ 0,25<br>(S)	0,1	≤ 1<br>(S)	≤ 0,25/<br>4,75<br>(S)
24	roe deer	*M. fortuitum*	2<br>(S)	16/8<br>(I)	8<br>(S)	≤ 4<br>(S)	≤ 4<br>(S)	≤ 0,12<br>(S)	0,12/<br>0,12<br>(S/S)	≤ 0,12<br>(S)	≤ 2<br>(S)	≤ 1<br>(S)	2<br>(I)	≤ 0,25<br>(S)	0,1	2<br>(S)	≤ 0,25/<br>4,75<br>(S)
25	roe deer	*M. fortuitum*	≤ 1<br>(S)	32/16<br>(R)	32<br>(R)	≤ 4<br>(S)	> 64<br>(R)	≤ 0,12<br>(S)	0,12/<br>0,12<br>(S/S)	4<br>(I)	≤ 2<br>(S)	2<br>(S)	2<br>(I)	≤ 0,25<br>(S)	0,1	2<br>(S)	≤ 0,25/<br>4,75<br>(S)
26	roe deer	*M. fortuitum*	≤ 1<br>(S)	32/16<br>(R)	> 32<br>(R)	8<br>(S)	> 64<br>(R)	0,5<br>(S)	≤ 0,06/<br>2<br>(S/S)	0,25<br>(S)	NR	≤ 1<br>(S)	≤ 1<br>(S)	≤ 0,25<br>(S)	0,5	2<br>(S)	≤ 0,25/<br>4,75<br>(S)
27	roe deer	*M. fortuitum*	≤ 1<br>(S)	32/16<br>(R)	16<br>(I)	≤ 4<br>(S)	> 64<br>(R)	≤ 0,12<br>(S)	≤ 0,06/<br>≤ 0,06<br>(S/S)	2<br>(I)	≤ 2<br>(S)	≤ 1<br>(S)	≤ 1<br>(S)	≤ 0,25<br>(S)	0	2<br>(S)	≤ 0,25/<br>4,75<br>(S)
28	roe deer	*M. fortuitum*	≤ 1<br>(S)	16/8<br>(I)	> 32<br>(R)	4<br>(S)	> 64<br>(R)	≤ 0,12<br>(S)	0,5/<br>0,5	4<br>(I)	≤ 2<br>(S)	2<br>(S)	4<br>(I)	≤ 0,25<br>(S)	0,1	2<br>(S)	1/19<br>(S)

No. in list	Animal species	NTM species	Interpretation of susceptibility test and MIC (µg/ml)														
			AMI	AUG2	FEP	FOX	AXO	CIP	CLA (5th day/ 14th day)	DOX	IMI	LZD	MIN	MXF	TGC	TOB	SXT
									(S/S)								
29	roe deer	*M. fortuitum*	≤ 1 (S)	16/8 (I)	> 32 (R)	≤ 4 (S)	> 64 (R)	≤ 0,12 (S)	4/4 (I/I)	2 (I)	≤ 2 (S)	≤ 1 (S)	≤ 1 (S)	≤ 0,25 (S)	0,5	≤ 1 (S)	≤ 0,25/ 4,75 (S)
No. in list	Animal species	NTM species	Interpretation of susceptibility test and MIC (µg/ml)														
			AMI	AUG2	FEP	FOX	AXO	CIP	CLA (5th day/ 14th day)	DOX	IMI	LZD	MIN	MXF	TGC	TOB	SXT
30	roe deer	*M. fortuitum*	≤ 1 (S)	64/32 (R)	> 32 (R)	8 (S)	> 64 (R)	≤ 0,12 (S)	≤ 0,06/ ≤ 0,06 (S/S)	4 (I)	≤ 2 (S)	4 (S)	2 (I)	≤ 0,25 (S)	0,1	2 (S)	0,5/9,5 (S)
31	roe deer	*M. fortuitum*	≤ 1 (S)	32/16 (R)	> 32 (R)	32 (I)	64 (R)	≤ 0,12 (S)	4/ > 16 (I/R)	16 (R)	NR	4 (S)	8 (R)	≤ 0,25 (S)	0,5	≤ 1 (S)	0,5/9,5 (S)
32	roe deer	*M. fortuitum*	≤ 1 (S)	32/16 (R)	> 32 (R)	8 (S)	> 64 (R)	≤ 0,12 (S)	≤ 0,06/ ≤ 0,06 (S/S)	8 (R)	4 (S)	2 (S)	4 (I)	≤ 0,25 (S)	0,1	2 (S)	≤ 0,25/ 4,75 (S)
33	roe deer	*M. fortuitum*	≤ 1 (S)	>64/32 (R)	> 32 (R)	16 (S)	> 64 (R)	≤ 0,12 (S)	≤ 0,06/ 0,25 (S/S)	4 (I)	8 (I)	2 (S)	≤ 1 (S)	≤ 0,25 (S)	0,1	16 (R)	0,5/9,5 (S)
34	roe deer	*M. fortuitum*	2 (S)	64/32 (R)	> 32 (R)	16 (S)	> 64 (R)	≤ 0,12 (S)	2/8 (S/R)	4 (I)	4 (S)	≤ 1 (S)	2 (I)	≤ 0,25 (S)	0,3	> 16 (R)	0,5/9,5 (S)
35	roe deer	*M. fortuitum*	≤ 1 (S)	32/16 (R)	> 32 (R)	8 (S)	> 64 (R)	≤ 0,12 (S)	≤ 0,06/ ≤ 0,06 (S/S)	4 (I)	4 (S)	2 (S)	1 (S)	≤ 0,25 (S)	0,1	2 (S)	0,5/9,5 (S)
36	roe deer	*M. fortuitum*	≤ 1 (S)	32/16 (R)	16 (I)	32 (I)	> 64 (R)	≤ 0,12 (S)	8/ > 16 (R/R)	16 (R)	NR	8 (S)	8 (R)	≤ 0,25 (S)	0,1	16 (R)	0,5/9,5 (S)
37	sheep	*M. neoaurum*	≤ 1 (S)	≤ 2/1 (S)	> 32 (R)	8 (S)	32 (I)	≤ 0,12 (S)	0,12/ 0,12 (S/S)	≤ 0,12 (S)	≤ 2 (S)	≤ 1 (S)	≤ 1 (S)	≤ 0,25 (S)	0,1	≤ 1 (S)	0,5/9,5 (S)
No. in list	Animal species	NTM species	Interpretation of susceptibility test and MIC (µg/ml)														
			AMI	AUG2	FEP	FOX	AXO	CIP	CLA (5th day/ 14th day)	DOX	IMI	LZD	MIN	MXF	TGC	TOB	SXT
38	chicken	*M. neoaurum*	≤ 1 (S)	≤ 2/1 (S)	16 (I)	8 (S)	16 (I)	≤ 0,12 (S)	≤ 0,06/ ≤ 0,06 (S/S)	≤ 0,12 (S)	≤ 2 (S)	≤ 1 (S)	≤ 1 (S)	≤ 0,25 (S)	0,1	≤ 1 (S)	≤ 0,25/ 4,75 (S)
39	wild boar	*M. neoaurum*	≤ 1 (S)	4/2 (S)	> 32 (R)	8 (S)	64 (R)	≤ 0,12 (S)	0,25/ 0,25 (S/S)	≤ 0,12 (S)	4 (S)	≤ 1 (S)	≤ 1 (S)	≤ 0,25 (S)	0,3	≤ 1 (S)	≤ 0,25/ 4,75 (S)
40	wild boar	*M. neoaurum*	≤ 1 (S)	≤ 2/1 (S)	16 (I)	8 (S)	> 64 (R)	≤ 0,12 (S)	0,25/ 0,25 (S/S)	≤ 0,12 (S)	16 (I)	≤ 1 (S)	≤ 1 (S)	≤ 0,25 (S)	0,3	2 (S)	≤ 0,25/ 4,75 (S)
41	wild boar	*M. neoaurum*	≤ 1 (S)	≤ 2/1 (S)	16 (I)	≤ 4 (S)	32 (I)	≤ 0,12 (S)	0,12/ 0,12 (S/S)	≤ 0,12 (S)	4 (S)	≤ 1 (S)	≤ 1 (S)	≤ 0,25 (S)	0,1	≤ 1 (S)	≤ 0,25/ 4,75 (S)
42	roe deer	*M. neoaurum*	≤ 1 (S)	≤ 2/1 (S)	32 (R)	≤ 4 (S)	8 (S)	≤ 0,12 (S)	0,25/ 0,25 (S/S)	≤ 0,12 (S)	≤ 2 (S)	≤ 1 (S)	≤ 1 (S)	≤ 0,25 (S)	0,3	≤ 1 (S)	≤ 0,25/ 4,75 (S)
43	roe deer	*M. neoaurum*	≤ 1 (S)	4/2 (S)	> 32 (R)	8 (S)	64 (R)	≤ 0,12 (S)	0,12/ 0,12 (S/S)	≤ 0,12 (S)	≤ 2 (S)	≤ 1 (S)	≤ 1 (S)	≤ 0,25 (S)	0,3	≤ 1 (S)	≤ 0,25/ 4,75 (S)
44	roe deer	*M. neoaurum*	≤ 1 (S)	4/2 (S)	> 32 (R)	8 (S)	32 (I)	≤ 0,12 (S)	0,12/ 0,12 (S/S)	≤ 0,12 (S)	8 (I)	≤ 1 (S)	≤ 1 (S)	≤ 0,25 (S)	0,3	≤ 1 (S)	≤ 0,25/ 4,75 (S)
No. in list	Animal species	NTM species	Interpretation of susceptibility test and MIC (µg/ml)														
			AMI	AUG2	FEP	FOX	AXO	CIP	CLA (5th day/	DOX	IMI	LZD	MIN	MXF	TGC	TOB	SXT

No. in list	Animal species	NTM species	Interpretation of susceptibility test and MIC (µg/ml)														
			AMI	AUG2	FEP	FOX	AXO	CIP	CLA (5th day/ 14th day)	DOX	IMI	LZD	MIN	MXF	TGC	TOB	SXT
									14th day)								
45	roe deer	*M. neoaurum*	≤ 1 **(S)**	4/2 **(S)**	32 **(R)**	8 **(S)**	64 **(R)**	≤ 0,12 **(S)**	0,25/ 0,25 **(S/S)**	≤ 0,12 **(S)**	16 **(I)**	≤ 1 **(S)**	≤ 1 **(S)**	≤ 0,25 **(S)**	0,3	≤ 1 **(S)**	≤ 0,25/ 4,75 **(S)**
46	roe deer	*M. neoaurum*	≤ 1 **(S)**	4/2 **(S)**	> 32 **(R)**	8 **(S)**	32 **(I)**	≤ 0,12 **(S)**	0,25/ 0,25 **(S/S)**	≤ 0,12 **(S)**	4 **(S)**	≤ 1 **(S)**	≤ 1 **(S)**	≤ 0,25 **(S)**	0,1	≤ 1 **(S)**	≤ 0,25/ 4,75 **(S)**
47	roe deer	*M. neoaurum*	≤ 1 **(S)**	8/4 **(S)**	> 32 **(R)**	8 **(S)**	64 **(R)**	≤ 0,12 **(S)**	0,25/ 0,25 **(S/S)**	≤ 0,12 **(S)**	4 **(S)**	≤ 1 **(S)**	≤ 1 **(S)**	≤ 0,25 **(S)**	0,3	≤ 1 **(S)**	≤ 0,25/ 4,75 **(S)**
48	roe deer	*M. neoaurum*	≤ 1 **(S)**	≤ 2/1 **(S)**	4 **(S)**	≤ 4 **(S)**	≤ 4 **(S)**	≤ 0,12 **(S)**	≤ 0,06/ ≤ 0,06 **(S/S)**	≤ 0,12 **(S)**	≤ 2 **(S)**	≤ 1 **(S)**	≤ 1 **(S)**	≤ 0,25 **(S)**	0,1	≤ 1 **(S)**	≤ 0,25/ 4,75 **(S)**
49	roe deer	*M. neoaurum*	≤ 1 **(S)**	≤ 2/1 **(S)**	32 **(R)**	≤ 4 **(S)**	32 **(I)**	≤ 0,12 **(S)**	0,12/ 0,12 **(S/S)**	≤ 0,12 **(S)**	≤ 2 **(S)**	≤ 1 **(S)**	≤ 1 **(S)**	≤ 0,25 **(S)**	0,3	≤ 1 **(S)**	≤ 0,25/ 4,75 **(S)**
50	roe deer	*M. neoaurum*	≤ 1 **(S)**	≤ 2/1 **(S)**	> 32 **(R)**	8 **(S)**	32 **(I)**	≤ 0,12 **(S)**	≤ 0,06/ ≤ 0,06 **(S/S)**	≤ 0,12 **(S)**	≤ 2 **(S)**	≤ 1 **(S)**	≤ 1 **(S)**	≤ 0,25 **(S)**	0,1	≤ 1 **(S)**	≤ 0,25/ 4,75 **(S)**
51	roe deer	*M. neoaurum*	≤ 1 **(S)**	≤ 2/1 **(S)**	16 **(I)**	8 **(S)**	16 **(I)**	≤ 0,12 **(S)**	≤ 0,06/ 0,12 **(S/S)**	≤ 0,12 **(S)**	≤ 2 **(S)**	≤ 1 **(S)**	≤ 1 **(S)**	≤ 0,25 **(S)**	0,1	≤ 1 **(S)**	≤ 0,25/ 4,75 **(S)**
No. in list	Animal species	NTM species	Interpretation of susceptibility test and MIC (µg/ml)														
			AMI	AUG2	FEP	FOX	AXO	CIP	CLA (5th day/ 14th day)	DOX	IMI	LZD	MIN	MXF	TGC	TOB	SXT
52	roe deer	*M. neoaurum*	≤ 1 **(S)**	≤ 2/1 **(S)**	> 32 **(R)**	≤ 4 **(S)**	32 **(I)**	≤ 0,12 **(S)**	0,12/ 0,25 **(S/S)**	≤ 0,12 **(S)**	≤ 2 **(S)**	4 **(S)**	≤ 1 **(S)**	≤ 0,25 **(S)**	0,1	8 **(R)**	≤ 0,25/ 4,75 **(S)**
53	roe deer	*M. neoaurum*	≤ 1 **(S)**	≤ 2/1 **(S)**	> 32 **(R)**	8 **(S)**	32 **(I)**	≤ 0,12 **(S)**	0,12/ 0,12 **(S/S)**	≤ 0,12 **(S)**	≤ 2 **(S)**	≤ 1 **(S)**	≤ 1 **(S)**	≤ 0,25 **(S)**	0,1	≤ 1 **(S)**	≤ 0,25/ 4,75 **(S)**
54	roe deer	*M. neoaurum*	≤ 1 **(S)**	≤ 2/1 **(S)**	16 **(I)**	≤ 4 **(S)**	8 **(S)**	≤ 0,12 **(S)**	0,12/ 0,12 **(S/S)**	≤ 0,12 **(S)**	≤ 2 **(S)**	≤ 1 **(S)**	≤ 1 **(S)**	≤ 0,25 **(S)**	0,1	≤ 1 **(S)**	≤ 0,25/ 4,75 **(S)**
55	roe deer	*M. neoaurum*	≤ 1 **(S)**	≤ 2/1 **(S)**	32 **(R)**	8 **(S)**	16 **(I)**	≤ 0,12 **(S)**	0,25/ 0,25 **(S/S)**	≤ 0,12 **(S)**	8 **(I)**	≤ 1 **(S)**	≤ 1 **(S)**	≤ 0,25 **(S)**	0,3	≤ 1 **(S)**	≤ 0,25/ 4,75 **(S)**
56	roe deer	*M. neoaurum*	≤ 1 **(S)**	≤ 2/1 **(S)**	≤ 1 **(S)**	8 **(S)**	≤ 4 **(S)**	≤ 0,12 **(S)**	≤ 0,06/ ≤ 0,06 **(S/S)**	≤ 0,12 **(S)**	≤ 2 **(S)**	≤ 1 **(S)**	≤ 1 **(S)**	≤ 0,25 **(S)**	0,3	≤ 1 **(S)**	0,5/9,5 **(S)**
57	roe deer	*M. neoaurum*	≤ 1 **(S)**	4/2 **(S)**	> 32 **(R)**	≤ 4 **(S)**	32 **(I)**	≤ 0,12 **(S)**	0,12/ 0,12 **(S/S)**	≤ 0,12 **(S)**	4 **(S)**	≤ 1 **(S)**	≤ 1 **(S)**	≤ 0,25 **(S)**	0,3	≤ 1 **(S)**	≤ 0,25/ 4,75 **(S)**
No. in list	Animal species	NTM species	Interpretation of susceptibility test and MIC (µg/ml)														
			AMI	AUG2	FEP	FOX	AXO	CIP	CLA (5th day/ 14th day)	DOX	IMI	LZD	MIN	MXF	TGC	TOB	SXT
58	roe deer	*M. neoaurum*	≤ 1 **(S)**	4/2 **(S)**	32 **(R)**	8 **(S)**	8 **(S)**	≤ 0,12 **(S)**	0,12/ 0,12 **(S/S)**	≤ 0,12 **(S)**	≤ 2 **(S)**	≤ 1 **(S)**	≤ 1 **(S)**	≤ 0,25 **(S)**	0,1	≤ 1 **(S)**	≤ 0,25/ 4,75 **(S)**
59	roe deer	*M. neoaurum*	≤ 1 **(S)**	≤ 2/1 **(S)**	32 **(R)**	≤ 4 **(S)**	16 **(I)**	≤ 0,12 **(S)**	0,12/ 0,12 **(S/S)**	≤ 0,12 **(S)**	≤ 2 **(S)**	≤ 1 **(S)**	≤ 1 **(S)**	≤ 0,25 **(S)**	0,1	≤ 1 **(S)**	≤ 0,25/ 4,75 **(S)**
60	roe deer	*M. neoaurum*	≤ 1 **(S)**	64/32 **(R)**	4 **(S)**	>128 **(R)**	32 **(I)**	≤ 0,12 **(S)**	16/16 **(R/R)**	1 **(S)**	≤ 2 **(S)**	> 32 **(R)**	≤ 1 **(S)**	≤ 0,25 **(S)**	0,5	≤ 1 **(S)**	8/152 **(R)**
61	roe deer	*M. neoaurum*	8 **(S)**	≤ 2/1 **(S)**	> 32 **(R)**	128 **(R)**	> 64 **(R)**	0,5 **(S)**	≤ 0,06/ ≤ 0,06 **(S/S)**	0,25 **(S)**	≤ 2 **(S)**	2 **(S)**	≤ 1 **(S)**	≤ 0,25 **(S)**	0	4 **(I)**	≤ 0,25/ 4,75 **(S)**

**No. in list**	**Animal species**	**NTM species**	**Interpretation of susceptibility test and MIC (µg/ml)**														
			AMI	AUG2	FEP	FOX	AXO	CIP	CLA (5th day/ 14th day)	DOX	IMI	LZD	MIN	MXF	TGC	TOB	SXT
62	roe deer	*M. peregrinum*	≤ 1 **(S)**	>64/32 **(R)**	> 32 **(R)**	16 **(S)**	> 64 **(R)**	≤ 0,12 **(S)**	(5th day) **(S)**	4 **(I)**	NR	4 **(S)**	4 **(I)**	≤ 0,25 **(S)**	0,3	8 **(R)**	0,5/ 9,5 **(S)**
63	wild boar	*M. phocaicum*	≤ 1 **(S)**	16/8 **(I)**	4 **(S)**	8 **(S)**	≤ 4 **(S)**	≤ 0,12 **(S)**	(5th day) **(S)**	0,25 **(S)**	NR	≤ 1 **(S)**	≤ 1 **(S)**	≤ 0,25 **(S)**	0,1	≤ 1 **(S)**	≤ 0,25/ 4,75 **(S)**
64	wild boar	*M. phocaicum*	≤ 1 **(S)**	8/4 **(S)**	4 **(S)**	8 **(S)**	≤ 4 **(S)**	≤ 0,12 **(S)**	(5th day) **(S)**	0,25 **(S)**	NR	≤ 1 **(S)**	≤ 1 **(S)**	≤ 0,25 **(S)**	0,5	≤ 1 **(S)**	≤ 0,25/ 4,75 **(S)**
65	wild boar	*M. phocaicum*	≤ 1 **(S)**	32/16 **(R)**	8 **(S)**	≤ 4 **(S)**	16 **(I)**	≤ 0,12 **(S)**	(5th day) **(S)**	≤ 0,12 **(S)**	NR	2 **(S)**	2 **(I)**	≤ 0,25 **(S)**	0,3	≤ 1 **(S)**	0,5/ 9,5 **(S)**
**No. in list**	**Animal species**	**NTM species**	**Interpretation of susceptibility test and MIC (µg/ml)**														
			AMI	AUG2	FEP	FOX	AXO	CIP	CLA (5th day/ 14th day)	DOX	IMI	LZD	MIN	MXF	TGC	TOB	SXT
66	wild boar	*M. phocaicum*	≤ 1 **(S)**	16/8 **(I)**	16 **(I)**	8 **(S)**	≤ 4 **(S)**	≤ 0,12 **(S)**	(5th day) **(S)**	0,25 **(S)**	NR	≤ 1 **(S)**	2 **(I)**	≤ 0,25 **(S)**	0,5	≤ 1 **(S)**	1/19 **(S)**
67	wild boar	*M. phocaicum*	≤ 1 **(S)**	32/16 **(R)**	> 32 **(R)**	≤ 4 **(S)**	> 64 **(R)**	≤ 0,12 **(S)**	(5th day) **(S)**	≤ 0,12 **(S)**	4 **(S)**	≤ 1 **(S)**	≤ 1 **(S)**	≤ 0,25 **(S)**	≤ 0,015	≤ 1 **(S)**	2/38 **(S)**
68	wild boar	*M. phocaicum*	≤ 1 **(S)**	≤ 2/1 **(S)**	8 **(S)**	≤ 4 **(S)**	≤ 4 **(S)**	≤ 0,12 **(S)**	(5th day) **(S)**	≤ 0,12 **(S)**	≤ 2 **(S)**	≤ 1 **(S)**	≤ 1 **(S)**	≤ 0,25 **(S)**	0,1	≤ 1 **(S)**	≤ 0,25/ 4,75 **(S)**
69	wild boar	*M. phocaicum*	≤ 1 **(S)**	≤ 2/1 **(S)**	4 **(S)**	8 **(S)**	8 **(S)**	0,25 **(S)**	(5th day) **(S)**	4 **(I)**	≤ 2 **(S)**	2 **(S)**	2 **(I)**	≤ 0,25 **(S)**	1	8 **(R)**	0,5/ 9,5 **(S)**
70	wild boar	*M. porcinum*	≤ 1 **(S)**	32/16 **(R)**	> 32 **(R)**	16 **(S)**	> 64 **(R)**	≤ 0,12 **(S)**	8/16 **(R/R)**	> 16 **(R)**	8 **(I)**	4 **(S)**	> 8 **(R)**	≤ 0,25 **(S)**	0,3	16 **(R)**	0,5/ 9,5 **(S)**
71	wild boar	*M. porcinum*	≤ 1 **(S)**	16/8 **(I)**	32 **(R)**	8 **(S)**	> 64 **(R)**	≤ 0,12 **(S)**	8/8 **(R/R)**	16 **(R)**	4 **(S)**	2 **(S)**	4 **(I)**	≤ 0,25 **(S)**	0,1	8 **(R)**	0,5/ 9,5 **(S)**
72	wild boar	*M. pulveris*	≤ 1 **(S)**	≤ 2/1 **(S)**	8 **(S)**	≤ 4 **(S)**	16 **(I)**	≤ 0,12 **(S)**	≤ 0,06/ ≤ 0,06 **(S/S)**	0,25 **(S)**	≤ 2 **(S)**	2 **(S)**	≤ 1 **(S)**	≤ 0,25 **(S)**	0,1	≤ 1 **(S)**	≤ 0,25/ 4,75 **(S)**
73	roe deer	*M. septicum*	≤ 1 **(S)**	4/2 **(S)**	4 **(S)**	≤ 4 **(S)**	8 **(S)**	≤ 0,12 **(S)**	≤ 0,06/ ≤ 0,06 **(S/S)**	2 **(I)**	8 **(I)**	2 **(S)**	≤ 1 **(S)**	≤ 0,25 **(S)**	0,1	≤ 1 **(S)**	≤ 0,25/ 4,75 **(S)**
**No. in list**	**Animal species**	**NTM species**	**Interpretation of susceptibility test and MIC (µg/ml)**														
			AMI	AUG2	FEP	FOX	AXO	CIP	CLA (5th day/ 14th day)	DOX	IMI	LZD	MIN	MXF	TGC	TOB	SXT
74	cattle	*M. vaccae*	≤ 1 **(S)**	≤ 2/1 **(S)**	> 32 **(R)**	8 **(S)**	32 **(I)**	≤ 0,12 **(S)**	1/1 **(S/S)**	≤ 0,12 **(S)**	≤ 2 **(S)**	≤ 1 **(S)**	≤ 1 **(S)**	≤ 0,25 **(S)**	0,1	≤ 1 **(S)**	0,5/ 9,5 **(S)**
75	cattle	*M. vaccae*	≤ 1 **(S)**	16/8 **(I)**	> 32 **(R)**	≤ 4 **(S)**	> 64 **(R)**	≤ 0,12 **(S)**	0,25/ 0,25 **(S/S)**	0,5 **(S)**	16 **(I)**	≤ 1 **(S)**	≤ 1 **(S)**	≤ 0,25 **(S)**	0,3	2 **(S)**	0,5/ 9,5 **(S)**
76	wild boar	*M. vaccae*	≤ 1 **(S)**	8/4 **(S)**	32 **(R)**	16 **(S)**	> 64 **(R)**	1 **(S)**	1/2 **(S/S)**	4 **(I)**	4 **(S)**	4 **(S)**	≤ 1 **(S)**	1 **(S)**	0,5	4 **(I)**	1/19 **(S)**
77	wild boar	*M. vaccae*	≤ 1 **(S)**	32/16 **(R)**	> 32 **(R)**	32 **(I)**	> 64 **(R)**	≤ 0,12 **(S)**	2/16 **(S/R)**	> 16 **(R)**	32 **(R)**	16 **(I)**	> 8 **(R)**	≤ 0,25 **(S)**	0,5	> 16 **(R)**	2/38 **(S)**
78	wild boar	*M. vaccae*	≤ 1 **(S)**	≤ 2/1 **(S)**	4 **(S)**	≤ 4 **(S)**	64 **(R)**	≤ 0,12 **(S)**	0,12/ 0,12 **(S/S)**	≤ 0,12 **(S)**	8 **(I)**	≤ 1 **(S)**	≤ 1 **(S)**	≤ 0,25 **(S)**	0,3	≤ 1 **(S)**	2/38 **(S)**
79	wild boar	*M. vaccae*	≤ 1 **(S)**	≤ 2/1 **(S)**	8 **(S)**	≤ 4 **(S)**	32 **(I)**	≤ 0,12 **(S)**	0,12/ 0,12 **(S/S)**	≤ 0,12 **(S)**	8 **(I)**	≤ 1 **(S)**	≤ 1 **(S)**	≤ 0,25 **(S)**	0,1	≤ 1 **(S)**	1/19 **(S)**

**No. in list**	**Animal species**	**NTM species**	**Interpretation of susceptibility test and MIC (μg/ml)**														
			AMI	AUG2	FEP	FOX	AXO	CIP	CLA (5th day/ 14th day)	DOX	IMI	LZD	MIN	MXF	TGC	TOB	SXT
80	wild boar	*M. vaccae*	≤1 **(S)**	>64/32 **(R)**	> 32 **(R)**	64 **(I)**	> 64 **(R)**	≤ 0,12 **(S)**	16/16 **(R/R)**	0,5 **(S)**	> 64 **(R)**	≤ 1 **(S)**	≤ 1 **(S)**	≤ 0,25 **(S)**	0,1	8 **(R)**	≤ 0,25/ 4,75 **(S)**
81	wild boar	*M. vaccae*	2 **(S)**	≤ 2/1 **(S)**	4 **(S)**	8 **(S)**	64 **(R)**	≤ 0,12 **(S)**	16/16 **(R/R)**	≤ 0,12 **(S)**	8 **(I)**	≤ 1 **(S)**	≤ 1 **(S)**	≤ 0,25 **(S)**	0,3	4 **(I)**	0,5/ 9,5 **(S)**
**No. in list**	**Animal species**	**NTM species**	**Interpretation of susceptibility test and MIC (μg/ml)**														
			AMI	AUG2	FEP	FOX	AXO	CIP	CLA (5th day/ 14th day)	DOX	IMI	LZD	MIN	MXF	TGC	TOB	SXT
82	wild boar	*M. vaccae*	≤1 **(S)**	≤ 2/1 **(S)**	4 **(S)**	8 **(S)**	64 **(R)**	≤ 0,12 **(S)**	> 16 (5th day) **(R)**	≤ 0,12 **(S)**	8 **(I)**	≤ 1 **(S)**	≤ 1 **(S)**	≤ 0,25 **(S)**	0,3	2 **(S)**	2/38 **(S)**
83	wild boar	*M. vaccae*	≤1 **(S)**	8/4 **(S)**	≤1 **(S)**	≤ 4 **(S)**	≤ 4 **(S)**	≤ 0,12 **(S)**	≤ 0,06/ ≤ 0,06 **(S/S)**	0,25 **(S)**	16 **(I)**	≤ 1 **(S)**	≤ 1 **(S)**	0,5 **(S)**	0,3	≤ 1 **(S)**	≤ 0,25/ 4,75 **(S)**
84	wild boar	*M. vaccae*	≤ 1 **(S)**	≤ 2/1 **(S)**	32 **(R)**	≤4 **(S)**	16 **(I)**	≤ 0,12 **(S)**	0,12/ 0,12 **(S/S)**	≤ 0,12 **(S)**	≤ 2 **(S)**	≤ 1 **(S)**	≤ 1 **(S)**	≤ 0,25 **(S)**	0,1	≤ 1 **(S)**	≤ 0,25/ 4,75 **(S)**
85	wild boar	*M. vaccae*	≤ 1 **(S)**	16/8 **(I)**	4 **(S)**	≤4 **(S)**	≤4 **(S)**	4 **(R)**	0,5/ 0,5 **(S/S)**	0,5 **(S)**	16 **(I)**	2 **(S)**	≤ 1 **(S)**	1 **(S)**	0,1	≤ 1 **(S)**	0,5/ 9,5 **(S)**
86	wild boar	*M. vaccae*	≤ 1 **(S)**	16/8 **(I)**	16 **(I)**	≤4 **(S)**	64 **(R)**	≤ 0,12 **(S)**	2/16 **(S/R)**	8 **(R)**	4 **(S)**	4 **(S)**	> 8 **(R)**	≤ 0,25 **(S)**	0,1	4 **(I)**	0,5/ 9,5 **(S)**
87	roe deer	*M. vaccae*	32 **(I)**	≤ 2/1 **(S)**	> 32 **(R)**	64 **(I)**	> 64 **(R)**	0,25 **(S)**	≤ 0,06/ ≤ 0,06 **(S/S)**	0,25 **(S)**	≤ 2 **(S)**	2 **(S)**	≤ 1 **(S)**	≤ 0,25 **(S)**	0,3	2 **(S)**	≤ 0,25/ 4,75 **(S)**
**No. in list**	**Animal species**	**NTM species**	**Interpretation of susceptibility test and MIC (μg/ml)**														
			AMI	AUG2	FEP	FOX	AXO	CIP	CLA (5th day/ 14th day)	DOX	IMI	LZD	MIN	MXF	TGC	TOB	SXT
	Positive control	*M. smegmatis* ATCC 19420	/	/	/	/	/	0,5	/	/	/	/	/	/	/	/	/

AMI, amikacin; ATCC, American Type Culture Collection; AUG2, amoxicillin/clavulanic acid, ratio 2:1; AXO, ceftriaxone; CIP, ciprofloxacin; CLA, clarithromycin; DOX, doxycycline; FEP, cefepime; FOX, cefoxitin; I, intermediate susceptible; IMI, imipenem; LZD, linezolid; MIC, minimum inhibitory concentration; MIN, minocycline; MXF, moxifloxacin; NR, not reported due to antibiotic instability; NTM, non-tuberculous mycobacteria; R, resistant; S, susceptible; SXT, trimethoprim – sulfamethoxazole; TGC, tigecycline; TOB, tobramycin.
